# Supplementary material for: Oral Administration of Omega-3 Fatty Acids Attenuates Lung Injury Caused by PM2.5 Respiratory Inhalation Simply and Feasibly In Vivo
Source: Int J Mol Sci. 2022 May 10;23(10):5323. doi: 10.3390/ijms23105323 (PMC9140442; doi:10.3390/ijms23105323)
Supplement: Supplementary file 1 [file ijms-23-05323-s001.zip › ijms-1667475-supplementary.pdf]

Supplementary materials

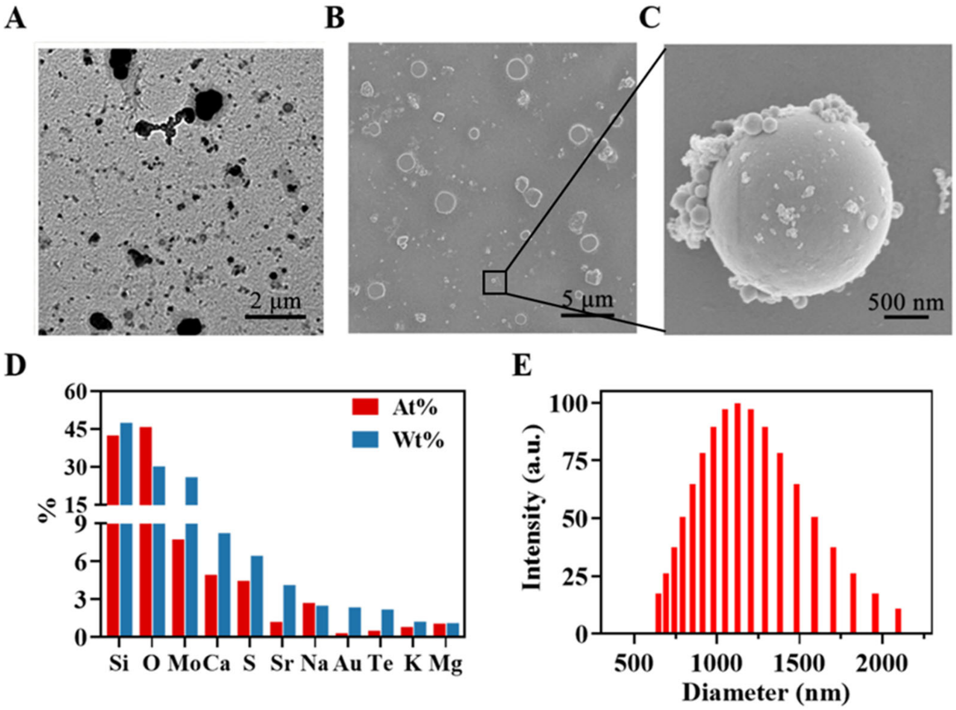

Figure S1. Characterization of PM 2.5. (A) Morphological characterization of water-insoluble PM 2.5. SEM images of (B) PM 2.5 and (C) representativePM 2.5 at higher maginifcation. (D) Atomic percentage and weight percentage of multiple elements in PM 2.5 detected by SEM-EDS. (E) The hydrodynamic size of PM 2.5 in deionized water.

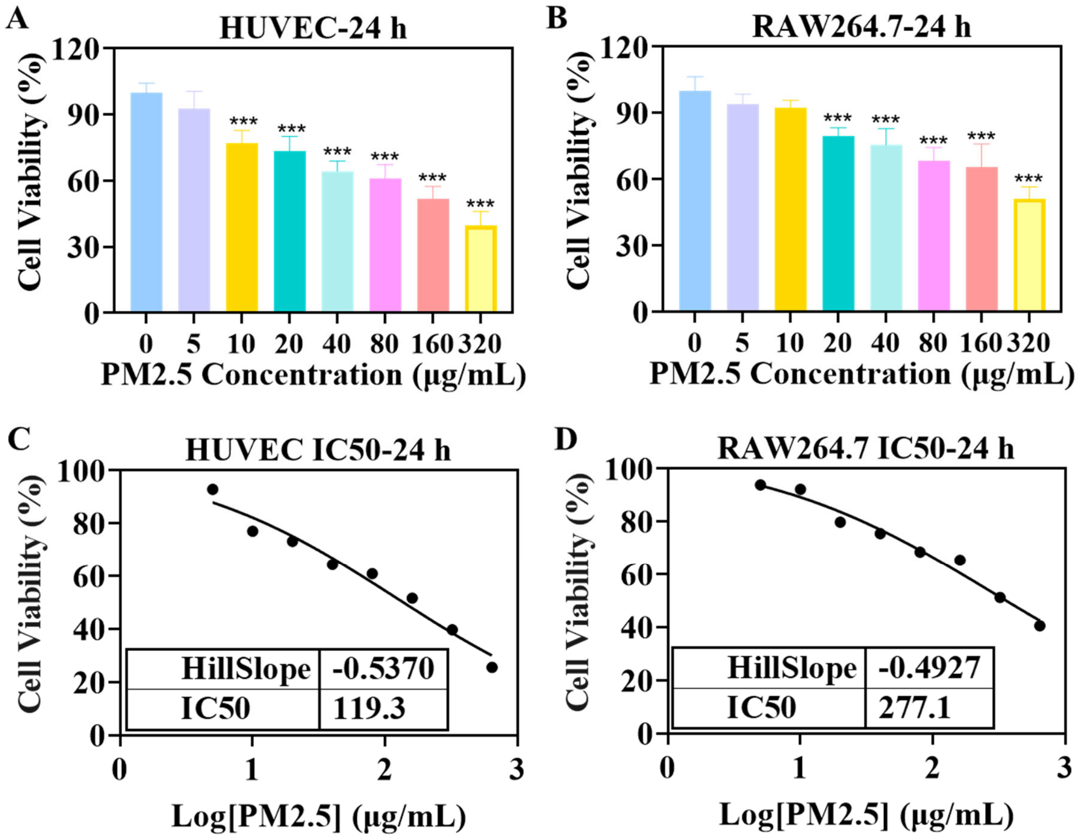

Figure S2. Cell viability of (A) HUVEC and (B) RAW264.7 cells treated with PM2.5 for 24 h. (C) The IC_{50} of HUVEC cells treated with PM2.5 for 24 h. (D) The IC_{50} of RAW264.7 cells treated with PM2.5 for 24 h. Data are expressed as the mean ± SD. *** P <0.001.

To further track PM 2.5 distribution *in vivo* and *in vitro*, Cy7-N-hydroxysuccinimide (Cy7, SE) fluorescent dye was covalently conjugated with these PM 2.5. As shown in Figure S2A, PM 2.5-Cy7 emits bright and stable red fluorescence (at 750 nm) in contrast to unlabeled PM 2.5. Infrared absorption peaks, CO-NH and C=O respectively are at 1650 cm^{-1} and 1726 cm^{-1}(Figure S1) confirmed conjugation of Cy7 onto the PM 2.5.

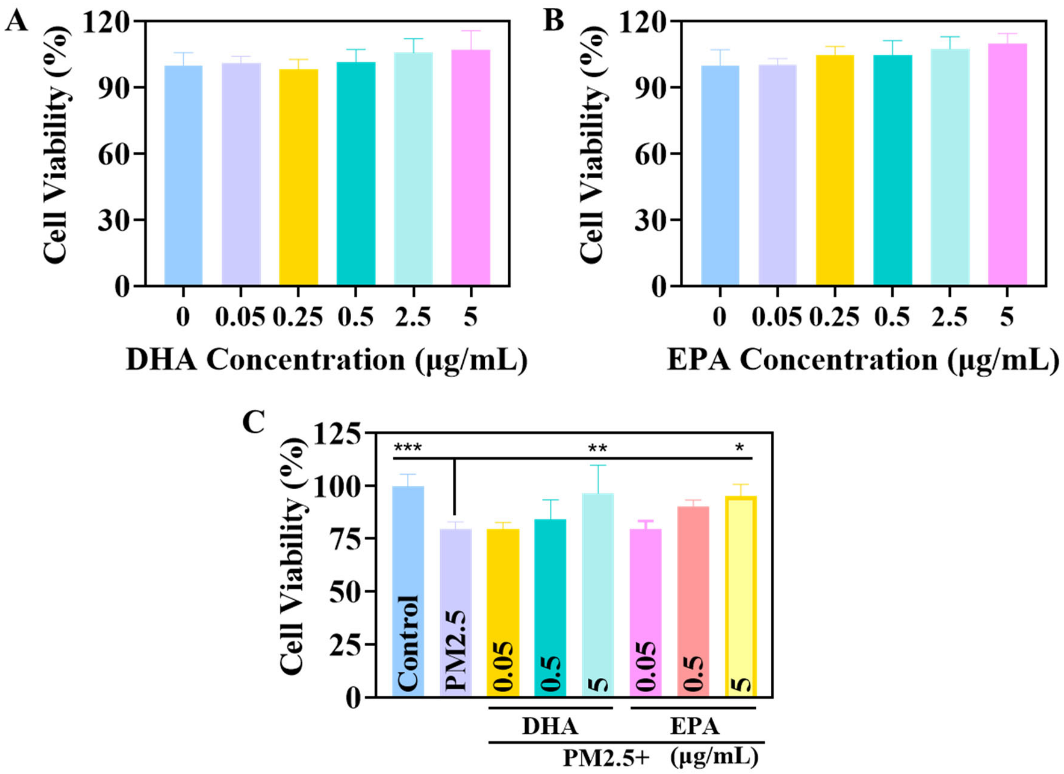

Figure S3. Effects of DHA and EPA on RAW264.7 cell activity. (A) Cell viability of RAW264.7 cells treated with DHA for 24 h. (B) Cell viability of RAW264.7 cells treated with EPA for 24 h. (C) Viability of RAW264.7 cells was determined after incubation with 0.05, 0.5, 5 μg/mL DHA or EPA and 20 μg/mL PM2.5 for 24 h. Data are expressed as the mean ± SD. *** P <0.001, ** P <0.01, * P<0.05.

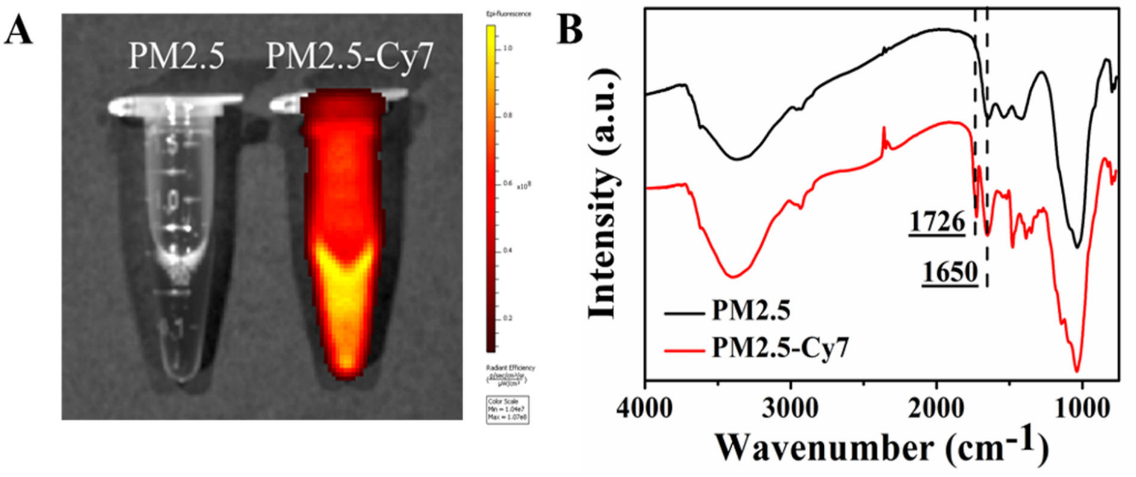

Figure S4. Fluorescent labeling of PM2.5. (A) Fluorescent image. (B) Infrared scanning spectrum of PM2.5 and PM2.5-Cy7.

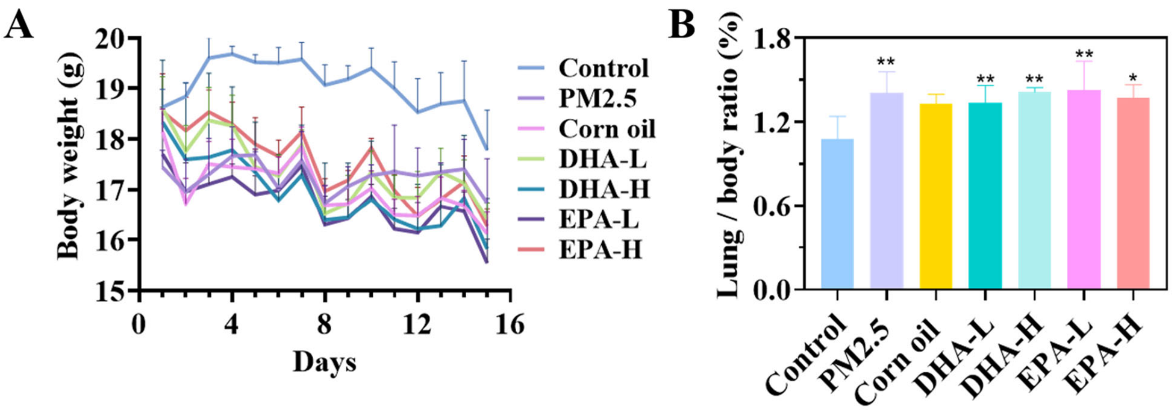

Figure S5. DHA and EPA ameliorated the toxicity of PM2.5 in mice model. (A) The body weight of seven groups. (B) The lung weight/body weight ratios in seven groups. Data are expressed as the mean ± SD. ** P <0.01, * P<0.05. The mice in control group were orally administered with saline.The mice in the corn oil group were orally administered with corn oil because corn oil was used to dissolve the DHA and EPA.

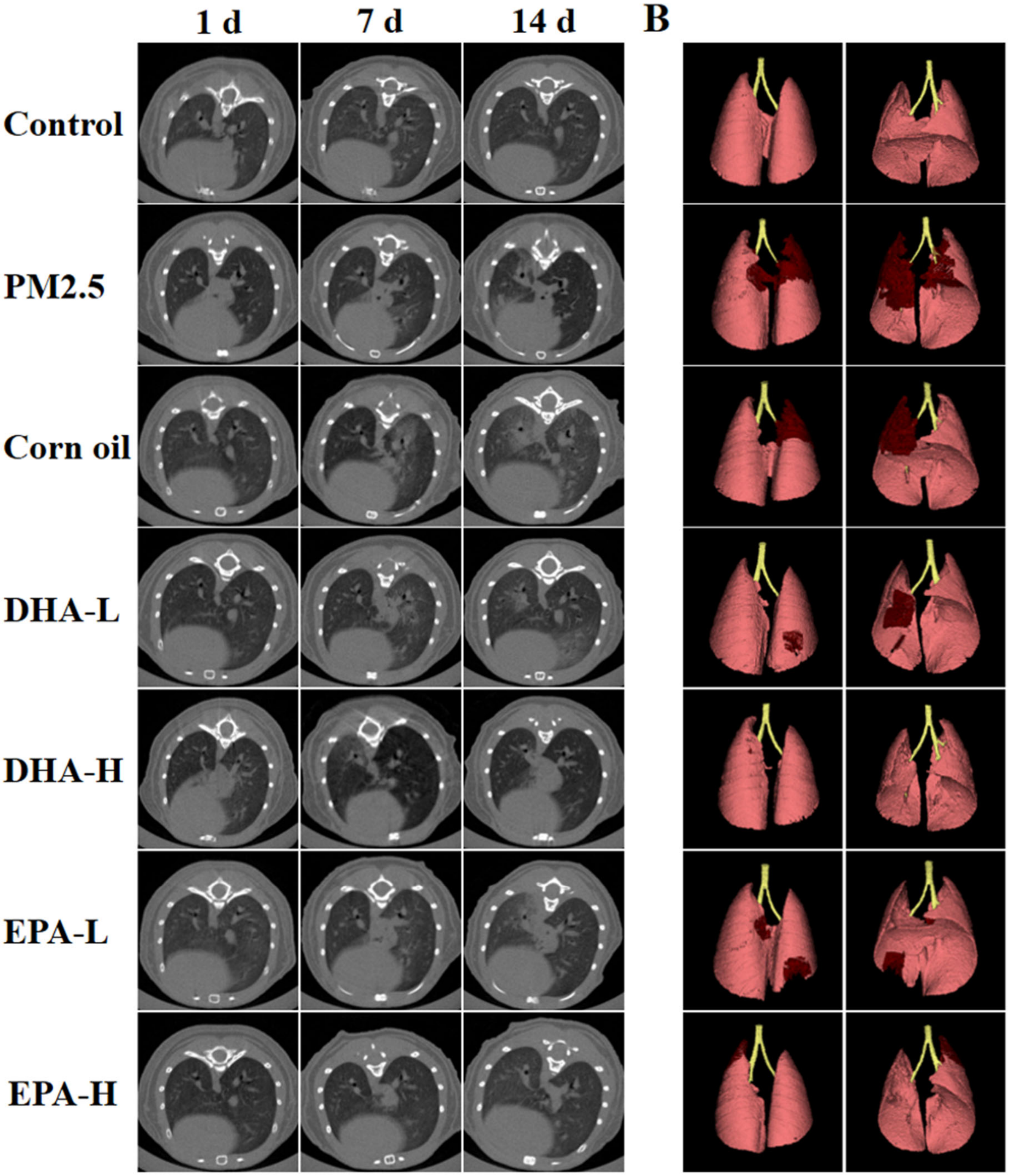

Figure S6. Micro-CT monitoring of lung pathogenesis 1 d, 7 d and 14 d after PM2.5 exposure. (B) Micro-CT three-dimensional reconstruction of lung tissue at the 14th day of PM2.5 exposure. The mice in control group were orally administered with saline.The mice in the corn oil group were orally administered with corn oil because corn oil was used to dissolve the DHA and EPA.

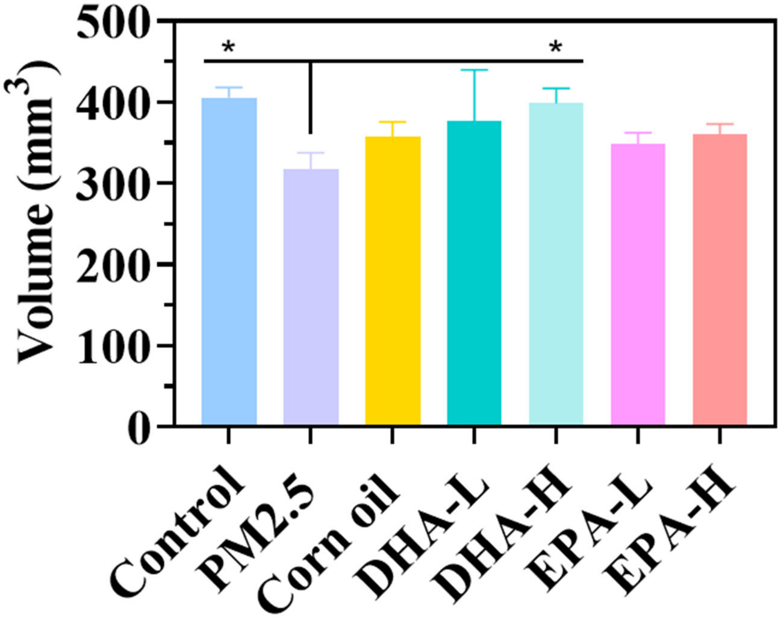

Figure S7. The volume of three-dimensional reconstruction of mice lung at the 14th day of PM2.5 exposure. Data are expressed as the mean ± SD. * P<0.05. The mice in control group were orally administered with saline.The mice in the corn oil group were orally administered with corn oil because corn oil was used to dissolve the DHA and EPA.

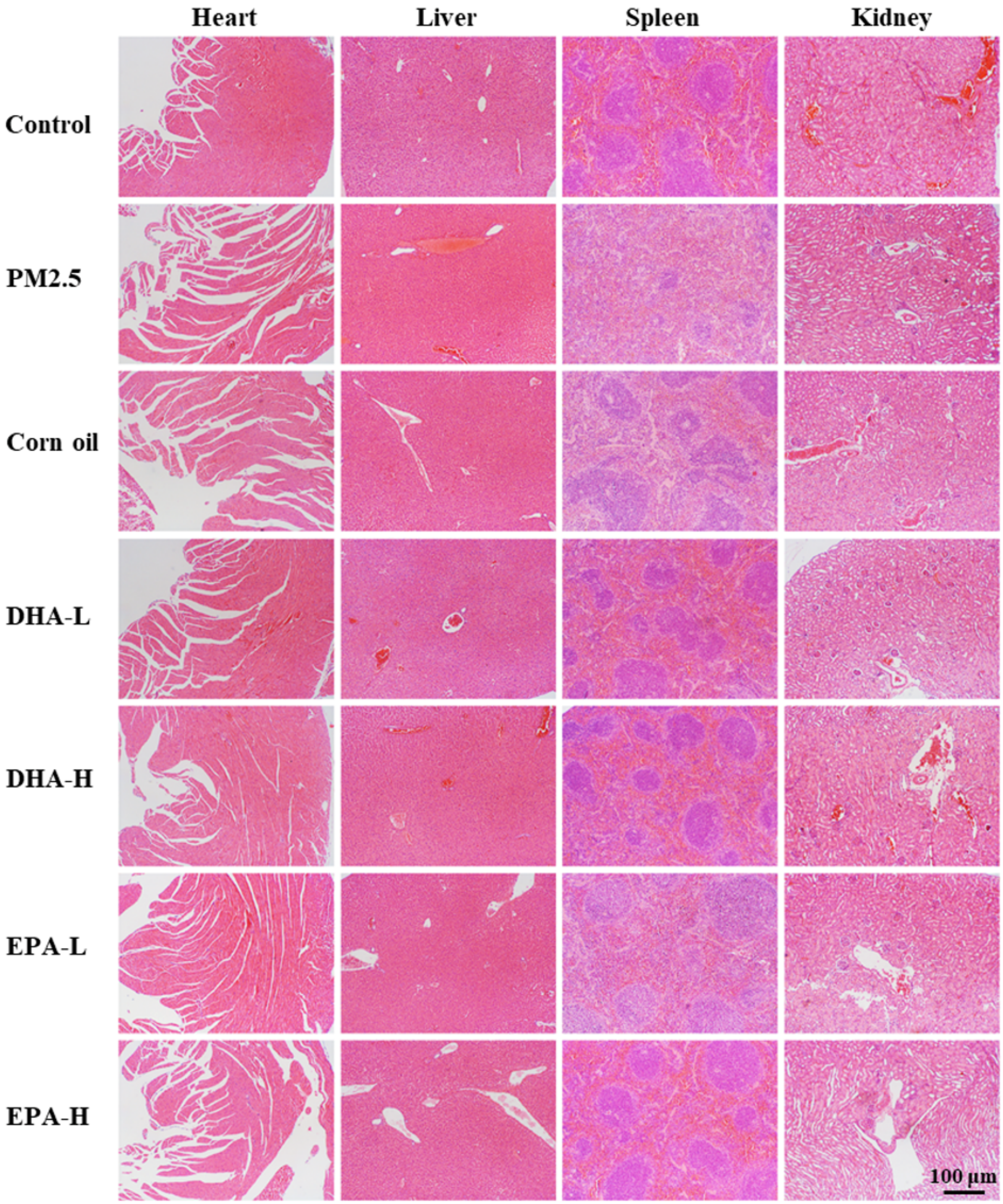

Figure S8. Histological examination of the heart, liver, spleen and kidney of mice induced by 4 times of PM2.5 and 14 days of DHA and EPA administration. The mice in control group were orally administered with saline.The mice in the corn oil group were orally administered with corn oil because corn oil was used to dissolve the DHA and EPA.

Table S1. A detailed diet composition.

	Basal
Ingredient (g)	
Soya protein, HD90	160
Met	3
Sucrose	638
Cellulose	50
Soyabean oil	100
Lard	10
Mineral mix	7
Calcium carbonate	10
Potassium phosphate monobasic	8
Potassium citrate	1.5
Vitamin mix	10
Choline bitartrate	2.5
Total	1000
Macronutrients (g)	
Protein	14.4
Carbohydrate	638
Fat	110
Fibre	50
Macronutrients (% of g)	
Protein	14.4
Carbohydrate	63.8
Fat	11
Fibre	5
Macronutrients (kcal)	
Protein	575
Carbohydrate	2552
Fat	990
Total	4117
Macronutrients(% of energy)	
Protein	14
Carbohydrate	62
Fat	24
Total	100
